# Hyaluronic Acid–Decorated Liposomes as Innovative Targeted Delivery System for Lung Fibrotic Cells

**DOI:** 10.3390/molecules24183291

**Published:** 2019-09-10

**Authors:** Laura Pandolfi, Vanessa Frangipane, Claudia Bocca, Alessandro Marengo, Erika Tarro Genta, Sara Bozzini, Monica Morosini, Maura D’Amato, Simone Vitulo, Manuela Monti, Giuditta Comolli, Maria Teresa Scupoli, Elias Fattal, Silvia Arpicco, Federica Meloni

**Affiliations:** 1 Research Laboratory of Lung Diseases, Section of Cell Biology, IRCCS Policlinico San Matteo Foundation, 27100 Pavia, Italy; 2 Department of Clinical and Biological Sciences, University of Turin, 10125 Turin, Italy; 3 Department of Drug Science and Technology, University of Turin, 10125 Turin, Italy; 4 Laboratory of Biotechnology, Center of Regenerative Medicine Research, IRCCS San Matteo Foundation, 27100 Pavia, Italy; 5 Experimental Research Laboratories, Biotechnology Area, IRCCS San Matteo Foundation, 27100 Pavia, Italy; 6 Molecular Virology Unit, Microbiology and Virology Department, IRCCS Policlinico San Matteo Foundation, 27100 Pavia, Italy; 7 Research Center LURM, Interdepartmental Laboratory of Medical Research, University of Verona, 37134 Verona, Italy; 8 Department of Neurosciences, Biomedicine and Movement Sciences, University of Verona, 37129 Verona, Italy; 9 Institut Galien Paris-Sud, CNRS, Université Paris-Sud, Université Paris-Saclay, 922996 Châtenay-Malabry, France; 10 Department of Internal Medicine, University of Pavia, 27100 Pavia, Italy

**Keywords:** liposomes, Bronchiolitis Obliterans Syndrome, lung fibrosis, hyaluronic acid, immune cells

## Abstract

Collagen Tissue Disease–associated Interstitial Lung Fibrosis (CTD-ILDs) and Bronchiolitis Obliterans Syndrome (BOS) represent severe lung fibrogenic disorders, characterized by fibro-proliferation with uncontrolled extracellular matrix deposition. Hyaluronic acid (HA) plays a key role in fibrosis with its specific receptor, CD44, overexpressed by CTD-ILD and BOS cells. The aim is to use HA-liposomes to develop an inhalatory treatment for these diseases. Liposomes with HA of two molecular weights were prepared and characterized. Targeting efficiency was assessed toward CTD-ILD and BOS cells by flow cytometry and confocal microscopy and immune modulation by RT-PCR and ELISA techniques. HA-liposomes were internalized by CTD-ILD and BOS cells expressing CD44, and this effect increased with higher HA MW. In THP-1 cells, HA-liposomes decreased pro-inflammatory cytokines IL-1β, IL-12, and anti-fibrotic VEGF transcripts but increased TGF-β mRNA. However, upon analyzing TGF-β release from healthy donors-derived monocytes, we found liposomes did not alter the release of active pro-fibrotic cytokine. All liposomes induced mild activation of neutrophils regardless of the presence of HA. HA liposomes could be also applied for lung fibrotic diseases, being endowed with low pro-inflammatory activity, and results confirmed that higher MW HA are associated to an increased targeting efficiency for CD44 expressing LFs-derived from BOS and CTD-ILD patients.

## 1. Introduction

Collagen Tissue Disease–associated Interstitial Lung Fibrosis (CTD-ILDs) and Bronchiolitis Obliterans Syndrome (BOS) represent two severe lung fibrogenic disorders involving, respectively, the lung interstitium and small airways, which share several pathogenic mechanisms and a poor long term outcome [1,2]. Common pathogenic steps include injury of airway epithelium due to immune-inflammatory insults, followed by a trigger of alveolar and bronchiolar epithelial cells transition towards myofibroblasts (epithelial mesenchymal transition, EMT) and a fibro-proliferative phase with uncontrolled extracellular matrix (ECM) deposition. Among ECM components (collagens, elastin, fibronectin, elastic fibers) [3], hyaluronic acid (HA) is the main one that characterizes fibrotic processes [4]. HA is a non-sulfated glycosaminoglycan synthesized by three transmembrane HA synthase (HAS1, HAS2, and HAS3) and binds specific protein partners: CD44 and the receptor for hyaluronan-mediated motility (RHAMM). Regarding CD44, the interaction with HA exerts a wide range of different physiological and pathological cell regulatory functions. In fibrosis, it has been demonstrated that HA enhances aberrant fibrotic cells motility through the binding with CD44 and RHAMM [5]. Moreover, Midgley et al. showed that EMT TGF-β-dependent of alveolar and bronchiolar epithelial cells was dependent upon HA/CD44/EGFR cascade [6].

We have recently demonstrated that primary lung fibroblasts (LFs) isolated from bronchoalveolar lavage (BAL) of CTD-ILD or BOS affected patients expressed a high rate of HA receptor CD44, and that this receptor represented a relevant and useful molecule with which to develop targeted nanovehicles [7]. In particular, we synthesized gold nanoparticles functionalized with a specific monoclonal antibody for CD44 in order to study their cytotoxicity onto LFs. We demonstrated that targeted nanovehicles efficiently decreased the viability of LFs in vitro, but in vivo analyses showed a trend of accumulation into alveolar macrophages after long-term inhalatory treatment of rodents [8]. As such, we decided to move toward more biocompatible nanocarriers (liposomes) decorated with a physiological targeting moiety. HA-functionalized nanocarriers are preferentially applied in cancer treatment [9,10,11,12], but given the important role of HA-CD44 interaction in tissues fibrosis progression, several research groups started to exploit HA-nanoparticles as drug delivery systems for liver fibrosis [13]. Regarding lung fibrosis, Chiesa et al. have already tried to exploit the properties of HA in drug delivery using chitosan nanoparticles, but theirs is the only research paper in this field [14].

Consequently, we planned to exploit the possibility of using HA-liposomes as vehicles to set the basis for an inhalatory treatment of CTD-ILD and BOS. To this aim, we decided to use two different low MW HA (4800 and 14,800 Da) for the preparation of liposomes, in order to explore, for the first time, the effect of HA MW on the targeting efficiency on lung fibrotic cells and inflammatory effectors. In particular, herein we describe the preparation, characterization, and biological properties evaluation of three different liposome preparations (LIP, LIP-HA4800, and LIP-HA14800) toward primary LFs derived from BOS or CTD-ILD affected patients and in the modulation of pro-inflammatory/fibrotic responses. Moreover, as our future perspective is the administration of HA-liposomes by an inhalatory route, we additionally assessed cell uptake on other lung cell types: human bronchial epithelial cell line (16-HBE), human alveolar basal epithelial cell line (A549), and mucus-producing bronchial epithelial cell line (Calu-3).

## 2. Results

### 2.1. Liposomes Formulation, and Characterization

LIP, LIP-HA4800, and LIP-HA14800, either blank and fluorescently labeled, were prepared by hydration of the lipid film followed by extrusion through polycarbonate filters to obtain homogenous small unilamellar vesicles. LIP-HA4800 and LIP-HA14800 were prepared by adding the HA4800-1,2-dipalmitoyl-sn-glycero-3-phosphoethanolamine (DPPE) or HA14800-DPPE conjugates during the hydration phase of lipid film: in this way, the phospholipidic chain was incorporated into the liposome membrane, while the HA was exposed toward the aqueous phase. For plain liposomes (LIP), l-α phosphatidyl-dl-glycerol sodium salt (PG) was used instead of HA-DPPE conjugates. Liposomes dimensions ranged from about 200 nm to 250 nm and the particle size of the HA-liposomes tended to increase with the increase of polymer MW, as previously reported [15]. The polydispersity index (PDI) was low for all the formulations (<0.18), and the zeta potential value was negative, for HA-liposomes the negative charge increased as the polymer MW increased due to the carboxylic negative residues of HA on the surface (Table S1).

### 2.2. Cells Internalization of Liposomes

**Figure 1:**
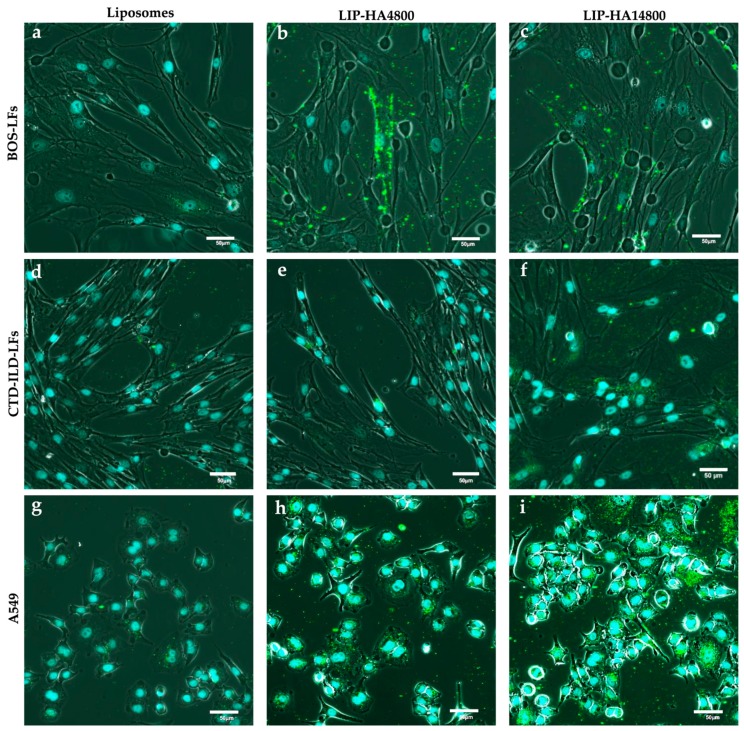
Confocal images of fluorescently labeled LIP (**a**,**d**,**g**), LIP-HA4800 (**b**,**e**,**h**) and LIP-HA14800 (**c**,**f**,**i**) and incubated with BOS-LFs (**a**–**c**), CTD-ILD-LFs (**d**–**f**) and A549 (**g**–**i**). Nuclei of cells = light blue (DAPI); liposomes = green signals. Scale bar = 50 μm.

In order to study the interactions of the three liposomal preparations with LFs derived from CTD-ILD and BOS, we firstly analyzed liposomes internalization by confocal microscopy, comparing results with A549 (CD44high), Calu-3 (CD44mild) 16-HBE (CD44neg) cell lines. Figure 1 shows that only HA-liposomes at both MW were internalized by BOS- (Figure 1a–c) and CTD-ILD- (Figure 1d–f) LF cells after 4 h of incubation. More internalization was observed upon incubating HA-liposomes with A549 with respect to LFs (Figure 1g–i), even if they had the same cell surface expression of CD44 (Table S2). As expected, we observed no significant interaction of LIP-HA at both MWs with 16-HBE (Figure S1a–c) and Calu-3 (Figure S1d–f), due to their poor expression of CD44 (Table S2). However, Figure S1f shows that only LIP-HA14800 were able to interact with Calu-3, although less than CD44high cells.

**Figure 2:**
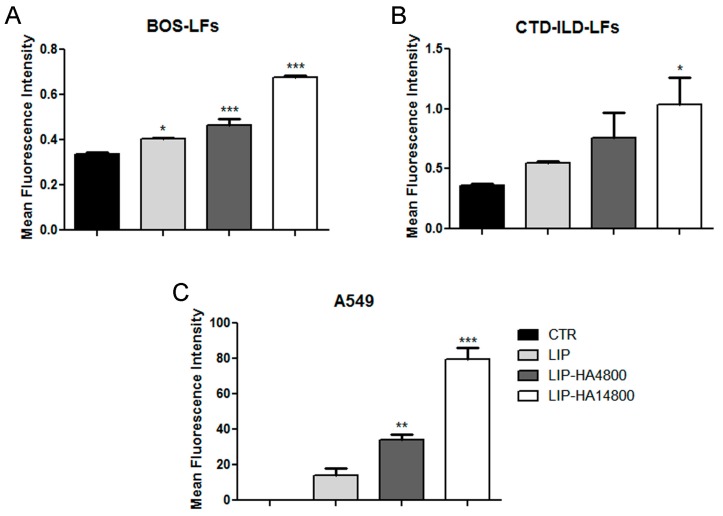
Flow cytometry analyses of fluorescently labeled LIP, LIP-HA4800, and LIP-HA14800 incubated with BOS-LFs (**A**), CTD-ILD-LFs (**B**), and A549 (**C**). Histograms represent mean of mean fluorescence intensity of liposomes ± standard deviation. *, *p* < 0.05 vs. CTR; **, *p* < 0.01 vs. CTR; ***, *p* < 0.001 vs. CTR.

To quantify the internalization of LIP, LIP-HA4800 and LIP-HA14800 by flow cytometry we decided to study only A549 and BOS- and CTD-ILD-LFs cells, since they demonstrated more significant results by confocal microscopy. As expected, we observed a higher interaction of LIP-HA with A549 (Figure 2C) rather than BOS- (Figure 2A) and CTD-ILD-LFs (Figure 2B), confirming confocal images (Figure 1). Moreover, thanks to this technique we understood that in all three cell lines the grade of liposomes cells uptake significantly increases with higher HA MW.

### 2.3. ECM Pericellular Coating

Knowing that BOS- and CTD-ILD-LFs produce a large amount of ECM, we wanted to further study whether the presence of ECM could be the cause of a different behavior of HA-liposomes interaction with these cells in comparison to A549 cells. Therefore, we co-cultured LFs or A549 with 1 × 10^8^ erythrocytes and confirmed that LFs release a pericellular ECM (Figure S2A) [4], as opposed to A549 (Figure S2B). Moreover, through incubating LFs with fluorescently labeled HA, which had been previously prepared in our laboratory, it was possible to observe a labeled-HA deposition as extracellular filamentous forms in LFs (Figure S3C) and not in A549 cells (Figure S2D), thus confirming the tendency of HA to interact with ECM produced by LFs in culture.

### 2.4. Liposomes Mucus Diffusion

**Figure 3:**
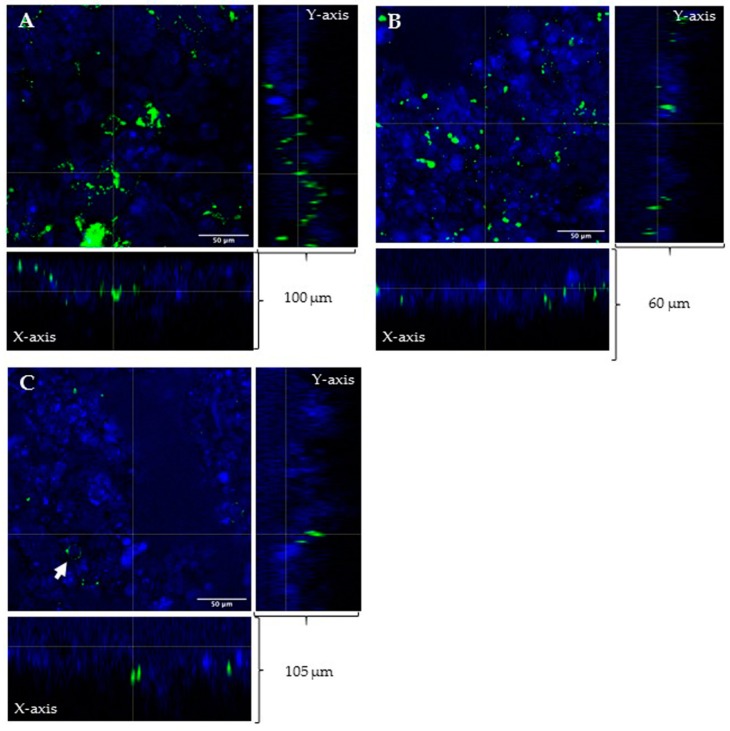
Confocal images of mucus layer diffusion of LIP (**A**), LIP-HA4800 (**B**), and LIP-HA14800 (**C**). (A–C) Cross-sectional profiles of the z-stack show green signals of liposomes and blue signals of DAPI (nuclei of cells) on both X and Y axis planes. LIP were found in all z-stacks (**A**). LIP-HA4800 (**B**) and LIP-HA14800 (**C**) were able to go deeper in the mucus layer. Arrow indicates interaction with Calu-3. Scale bar = 50 μm.

As our final goal is to administer liposomes through an inhalatory route, we wanted to study the interaction of three liposomal preparations with mucus layer coating respiratory epithelium. With this aim, we assessed mucus penetration on a 23-day culture of Calu-3 cells cultivated in air-liquid interface (ALI) configuration. The homogenous production of a mucus layer in these experimental conditions was previously assessed by alcian blue staining (Figure S3). With the aid of confocal microscopy, we were able to detect unfunctionalized liposomes in all sections made by z-stack (Figure 3A). On the contrary, HA decorated liposomes were found at deeper levels (only at the lower slice of z-stack: see Figure 3B,C) after the same incubation time. LIP-HA14800 were also able to reach cells and to interact with some of them (Figure 3C), confirming the results obtained in Figure S1f.

### 2.5. THP-1 Cell Uptake

**Figure 4:**
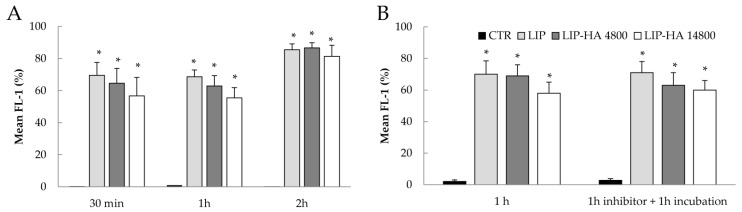
Cellular uptake of fluorescent LIP, LIP-HA4800, and LIP-HA14800 in THP-1 cells. Analysis of internalization of different liposomal formulations (**A**) and in presence of high MW HA conducted in THP-1 cells by flow cytometry after incubation for the indicated time (**B**). Histograms represent mean ± standard deviation expressed as percentage of fluorescence intensity of liposomes fluorescence signals of three independent experiments. *, *p* < 0.001 vs. CTR.

Efficient cellular uptake is a major requirement for the therapeutic efficacy of liposomes targeting, but in BOS and CTD-ILD, context is also important in order to consider the effect of different liposomal formulations on immune system modulation. Considering the important role of macrophages in lung fibrosis progression and the high expression of CD44 on their surface [16,17], we analyzed whether liposomes would be internalized by the human monocytic leukemia cell line (THP-1 cells) differentiated toward macrophages lineage with PMA. First, we confirmed the expression of CD44 on THP-1 by flow cytometry. Table S2 shows that both undifferentiated and differentiated THP-1 highly expressed CD44, and differentiation with PMA increased CD44 expression as reported in the literature [18]. Next, to analyze cellular uptake, we treated cells with fluorescently labeled LIP, LIP-HA4800, and LIP-HA14800 for the indicated times. We observed a rapid internalization of all liposomes, reaching plateau after 2 h, (approximately 85%, Figure 4A). In addition, the cellular uptake efficiency seems not to be dependent on the MW of HA. We investigated whether the observed liposomes uptake was mediated by cell surface CD44 receptor, and thus we pre-incubated differentiated THP-1 with a saturable amount of free high MW HA (51,000 Da) and subsequently with different liposomal formulations. These studies evidenced that the blockage of the receptor did not reduce the cellular uptake of liposomes, suggesting that in THP-1 cells the uptake is not CD44-dependent (Figure 4B), but rather is due to the phagocytic activity of this cell line.

### 2.6. Effect of Liposomes on Cytokines Expression

**Figure 5:**
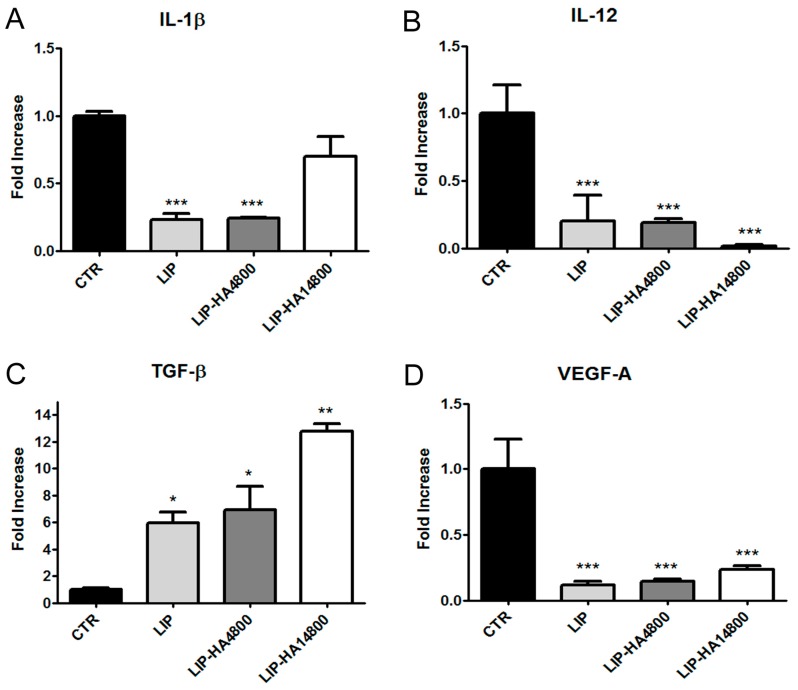
Analysis of transcript levels of IL-1β (**A**), IL-12 (**B**), TGF-β1 (**C**), and VEGF-A (**D**) in differentiated THP-1 cells. Quantitative real-time PCR (qPCR) of mRNAs of THP-1 cells treated for 1 h with LIP, LIP-HA4800, and LIP-HA14800. mRNA values are expressed as fold increase over control values after normalization to the GAPDH gene expression and are the means ± SEM of three independent experiments. *, *p* < 0.05, **, *p* < 0.01, ***, *p* < 0.001 vs. CTR.

Finally, we analyzed whether the different formulations of liposomes may modulate the activation of THP-1 cells. Specifically, we focused our attention on the determination of the transcript levels of well-known cytokines expressed by pro-inflammatory macrophages (M1) (IL-1β and IL-12) and cytokines correlated to the M2 phenotype (TGF-β, VEGF) involved in anti-inflammatory, pro-fibrotic, and pro-angiogenetic effects by quantitative real-time PCR (qRT-PCR) [19]. In our study, we found preliminarily that the exposure of differentiated human THP-1 cells to LIP, LIP-HA4800, and LIP-HA14800 significantly downregulated the transcript levels of the pro-inflammatory cytokines IL-1β and IL-12 with respect to the control cells (Figure 5A,B), while all formulations were able to induce the increase transcript levels of TGF-β cytokine (Figure 5C), with the highest effect occurring after incubation with LIP-HA14800. The pro-angiogenetic VEGF-A transcript level was significantly downregulated compared to control by all treatments (Figure 5D).

### 2.7. Effect of Liposomes on Monocytes and Neutrophils Derived From Blood of Healthy Donors

**Figure 6:**
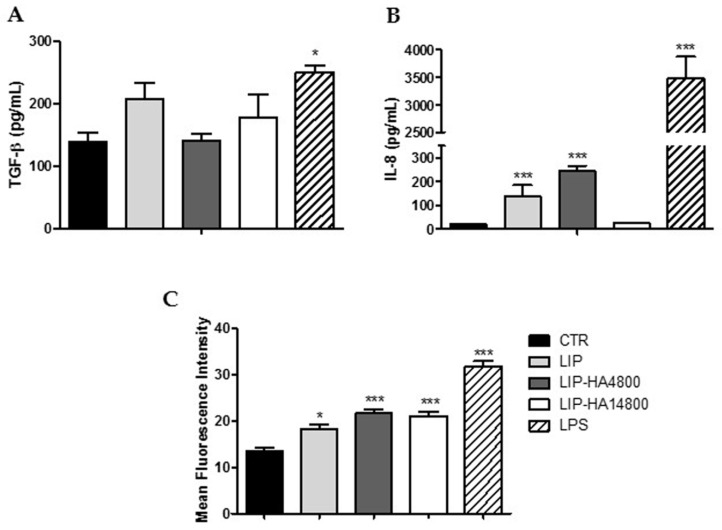
Liposomes biological effects on TGF-β (**A**) and IL-8 (**B**) release by monocytes isolated from peripheral blood. Analyses of CD11b surface expression on neutrophils by flow cytometer. All analyses were conducted after treating cells with LIP, LIP-HA4800 and LIP-HA14800 after 24 h for monocytes (**A**,**B**) and 4 h for neutrophils (**C**). LPS treatment was used as control. Data are represented as histograms of mean ± standard deviation. *, *p* < 0.05 vs. CTR; ***, *p* < 0.001 vs. CTR.

Even if the increase of TGF-β transcript in THP-1 differentiated cells induced by liposomes could be seen as anti-inflammatory activity, this cytokine plays a key role in fibrotic processes [19,20]. Hence, we decided to further study the effect of liposomes on monocytes isolated from the blood of healthy donors, analyzing the release of TGF-β and not the expression of mRNA, in order to have more reliable results on a physiologic cell line model. Figure 6A shows that treatment with all formulations did not significantly alter the release of pro-fibrotic cytokine from monocytes, compared to positive control treatment. Moreover, we assessed the ability of three preparations to modulate the release of a pro-inflammatory cytokine strongly related to neutrophilic inflammation, IL-8, from monocytes. LIP and LIP-HA4800 led to an increase of IL-8 release, rather than LIP-HA14800 (Figure 6B).

Since it has been demonstrated that both BOS and CTD-ILD start with an insult of lung epithelium by activated neutrophils, we decided to additionally analyze if liposomes were able to modulate this population. With this intent, we incubated neutrophils isolated from blood with LIP, LIP-HA4800, and LIP-HA14800, assessing the surface expression of CD11b, a surface marker of activation and degranulation [21]. Figure 6B shows that all formulations increased the surface expression of CD11b on neutrophils, but less than LPS positive control. Furthermore, since this effect was observed in liposomes decorated or not with HA, we can presume that the result was not due to the presence of HA but rather to the activation of neutrophils induced by liposome phagocytosis.

## 3. Discussion

HA is a physiologic glycoprotein used as moiety to functionalize nanocarriers to target cancer cells overexpressing CD44, one of the principal HA receptors. Since it has been reported that primary fibrotic cells express high rates of CD44 [7,9,22], researchers have started to exploit the same functionalization moiety to develop a targeted nano-based therapy for fibrosis. Regarding pulmonary fibrosis, only one paper has been published thus far describing the advantage of using HA conjugated onto nanoparticles [14]. With the work herein, we wanted to provide the basis for using HA as ligand to develop targeted nano-therapy against cells causing two lung fibrotic disorders—CTD-ILD and BOS. These severe diseases are associated to poor survival and as yet, treatment strategies are scarce, inadequate and associated to a significant systemic toxicity.

We have already studied and demonstrated that the use of drug-loaded gold nanoparticles functionalized with anti-CD44 antibody could be useful in inhibiting LFs proliferation inducing apoptosis [7]. However, in vivo experiments, aerosolizing nanoparticles to healthy mice, demonstrated that gold nanocarriers tended to accumulate inside lungs, mainly in alveolar macrophages. This observation raised the issue of a possible significant toxicity associated to long term inhalatory treatment. For this reason, we shifted our attention to more biocompatible nanovehicles, liposomes, due to their numerous reported advantages [23,24,25,26]. Our future goal is to deliver liposomes through the inhalation route in order to enhance therapeutic efficiency and avoid first-pass metabolism, and it has been demonstrated that this kind of nanoparticles are efficient in localizing the action of inhaled drugs in the lung. Moreover, they are already being used in clinical trials [26].

In the present study, we started to assess liposomes decorated with HA at two different MW. The choice of HA size is a crucial aspect due to the different activity of HA based on its MW [15]. In the literature, all MW of HA have been employed, from high MW to oligomers; herein, we decided to decorate liposomes with low MW HA (4800 and 14,800 Da). We were able to efficiently decorate liposome surfaces with both MW HA (mean particles size increased in correlation to MW of HA) with a good polydispersity index (<0.7) (Table S1) [27]. After liposomes characterization, we analyzed the targeting efficiency of LIP-HA4800 and LIP-HA14800 on LFs CTD-ILD and BOS-derived comparing results with liposomes without HA decoration (LIP). We thus demonstrated that HA is essential for internalization of liposomes. Moreover, the internalization is greater with HA14800 with respect to HA4800. This result is reasonable because HA is characterized by a repeated nature, so higher MW have more binding sites for CD44 [28,29]. However, we observed less internalization of LIP-HA with LFs as compared to A549 (positive control cell lines) (Figure 1 and Figure 2), even if they expressed the same quantity of surface receptor CD44 (Table S2). We subsequently analyzed the presence of pericellular coating, observing that only LFs derived from CTD-ILD and BOS showed this phenomenon due to their ability to produce ECM as fibrotic cells (Figure S3).

As our aim is the administration of HA-liposomes through the inhalation route, we assessed if the presence of HA onto liposomes could modulate the diffusion of nanovehicles into the mucus layer coating respiratory epithelium. For this purpose, we used an airway epithelium model (Calu-3) [30] in ALI configuration to allow the production of mucus. Following treatment with LIP, LIP-HA4800 and LIP-HA14800, we demonstrated that HA14800 increased the diffusion efficiency of liposomes in contrast to HA4800 (Figure 3). To the best of our knowledge, in the literature there are studies analyzing mucus penetrating ability of nanovehicles, correlating this activity to surface charge and to MW of polyethylene glycol (PEG) coating [31,32,33]. Very recently a research group modified lipoplexes (LX) for siRNA delivery to the lung with PEG and HA and demonstrated that non-PEGylated HA-LX diffused comparably to PEGylated LX, in contrast to non-modified LX, which remained entrapped in the mucus [33]. In this paper we observed the same result using liposomes without PEG modification: mucus penetration increased in the presence of HA.

Having assessed the behavior of liposome formulations with cells and mucus, we moved on to analyze whether nanovehicles were able to modulate immune cells activity, since BOS and CTD-ILD are due to a significant dysregulated local inflammatory response. Firstly, we used THP-1, a monocitic cell line, after differentiation induced by PMA. Analyses of THP-1 mRNA transcripts after treatment with LIP, LIP-HA4800, and LIP-HA14800 demonstrated that all treatments decreased the expression of IL-1β and IL-12, two relevant pro-inflammatory cytokines [34]. Since we are studying two lung fibrotic disorders, we decided to also analyze the VEGF and TGF-β mRNA levels due to their involvement in the pathogenesis of ILD [35]. All liposomal formulations led to a decrease of mRNA of VEGF (Figure 5D), while TGF-β expression increased after treatments, in particular with LIP-HA14800 (Figure 5C). This suggests that liposomes, in particular those decorated with HA14800, enhanced THP-1 PMA-induced toward an M2 phenotype, indirectly increasing the expression of pro-fibrogenic TGF-β. Although the THP-1 cell line is an established model for monocytic behavior and exerts many functions similar to blood monocytes, there exists some evidence indicating a significant divergence between the differentiation pattern of PMA stimulated THP-1 and peripheral monocytes [36]. Thus, we decided to assess liposomes modulation in the release of pro-inflammatory cytokine, IL-8 (strongly related to neutrophilic inflammation) and TGF-β in monocytes isolated from peripheral blood obtained from healthy donors. We demonstrated that only LIP-HA14800 did not alter the release of IL-8, but none of our liposomes were able to significantly alter TGF-β release (Figure 6A). The discrepancy regarding Figure 5C and Figure 6A can be explained by the neoplastic and poorly differentiated nature of THP-1 cells that need to be maximally induced by PMA treatment, altering the response to liposomes.

Finally, the neutrophils activation status was assessed by evaluating the CD11b surface expression level after liposomes treatment, since neutrophils are involved in early pathogenesis of CTD-ILD and BOS. Flow cytometry allowed us to determine that all formulations were capable of inducing a mild increase of CD11b expression level regardless of the presence of HA, even if significantly less than LPS positive control (Figure 6B). We assume that this result was due to the phagocytic nature of neutrophils.

In conclusion, we can infer that HA decoration of liposomes might be useful in the targeted treatment of lung fibrotic disorders, and, in addition, in our experimental models, we confirmed that a higher HA MW increases the CD44 targeting efficiency without a significant associated pro-inflammatory activity.

## 4. Materials and Methods

### 4.1. Materials and Instruments

Sodium hyaluronate of MW 4800 (HA4800) and 14,800 (HA14800) Da were purchased from Lifecore Biomedical (Chaska, MN, USA). All the phospholipids were provided by Avanti Polar-Lipids distributed by Sigma-Aldrich S.r.l. (Milan, Italy). Cholesterol, all the other chemicals, RPMI, amphotericin-B, and alcian blue were obtained from Sigma-Aldrich S.r.l. Fluorescein-5-(and-6)-sulfonic acid trisodium salt was purchased from Invitrogen, Life Technologies (Monza, Italy) Conjugates between HA and DPPE (HA-DPPE) were prepared using the method described in Arpicco et al. [14]. Dulbecco’s modified Eagle medium (DMEM), fetal calf serum (FCS), penicillin/streptomycin (P/S) solution, l-glutamine and phosphate buffer saline (PBS) were all purchased by Euroclone (Milan, Italy).

### 4.2. Preparation of Liposomes

Liposomes were prepared by thin lipid film hydration and extrusion method. Briefly, a chloroform solution of the lipid components 1,2-dipalmitoyl-*sn*-glycero-3-phosphocholine (DPPC), cholesterol (Chol), and PG (70:30:3 molar ratios) was evaporated and the resulting lipid film was dried under vacuum overnight. Lipid films were hydrated with HEPES [4-(2-hydroxyethyl) piperazine-1-ethanesulforic acid] buffer (pH 7.4), and the suspension was vortexed for 10 min and bath sonicated. The formulations were then sequentially extruded (Extruder, Lipex, Vancouver, BC, Canada) through a 400, and then a 200 nm polycarbonate membrane (Costar, Corning Incorporated, NY, USA) at a set temperature of 5 °C above the phase transition temperature of the lipid mixture. Fluorescently labeled liposomes were prepared as described above and a 10 mM solution of fluorescein-5-(and-6)-sulfonic acid trisodium salt in HEPES buffer was used during hydration. Liposomes were then purified through chromatography on Sepharose CL-4B columns, eluting with HEPES buffer at room temperature.

To prepare HA-liposomes, the same method of preparation was used and the lipid film was hydrated using a solution of the different HA-DPPE conjugates (three molar ratio) in HEPES buffer. Liposomes were stored at 4 °C.

### 4.3. Liposomes Characterization

The mean particle size and the PDI of liposomes were determined at 25 °C by quasi-elastic light scattering (QELS) using a nanosizer (Nanosizer Nano Z, Malvern Inst., Malvern, UK). The selected angle was 173° and the measurement was made after dilution of the liposome suspension in MilliQ^®^ water. Each measure was performed in triplicate. The particle surface charge of liposomes was investigated by zeta potential measurements at 25 °C applying the Smoluchowski equation and using the Nanosizer Nano Z. Measurements were carried out in triplicate. Phospholipid phosphorous was assessed in each liposome preparation by phosphate assay after destruction with perchloric acid [37].

### 4.4. Cell Lines

A549 (CD44high), Calu-3 (CD44mid) 16-HBE (CD44neg) cell lines (all purchased from ATCC^®^, Manassas, VA, USA) were cultivated in high glucose DMEM supplemented with 10% FCS, 100 U mL^–1^ P/S solution and 100 U mL^–1^ l-glutamine.

In order to allow the formation of a mucus layer, Calu-3 were cultured in ALI configuration: 1 × 10^6^ cells were seeded on the apical compartment of six-well transwell (0.4 µm pore size, Corning Costar, Lowell, MA, USA). The apical medium was removed after 24 h to allow the establishment of air-interface. Every two days, the medium on the apical side was eliminated and fresh medium was added only in the basal compartment until a complete layer of mucus was produced (day 23).

LFs were isolated from Bronchoalveolar Lavage (BAL) of patients affected by BOS and CTD-ILD obtained following standard recommendations [7]. 6 × 10^6^ cells were seeded in the same medium of cultivation. Single *foci* of LFs formed between 1–3 weeks were isolated and cultivated.

A549, Calu-3, 16-HBE and LFs were characterized for CD44 expression (using anti-CD44-FITC antibody, Beckman Coulter, Brea, CA, USA) by flow cytometer (Navios, Beckman Coulter, Brea, CA, USA).

Human monocytic leukemia cell line (THP-1 cells, purchased by ATCC^®^, Manassas, VA, USA) was maintained in RPMI medium supplemented with 10% FCS, 100 U mL^–1^ P/S and 25 μg mL^–1^ amphotericin-B. For all experiments with THP-1, 7 × 10^5^ cells were seeded in 35 mm petri dishes (Corning Costar, Lowell, MA, USA) and differentiated into macrophages-like phenotype with phorbol 12-myristate 13-acetate (PMA, 50 nM) for 48 h. After 24 h of incubation with fresh medium, THP-1 cells were then treated with LIP, LIP-HA4800, and LIP-HA14800.

### 4.5. Histological Staining for Mucus Production

Alcian blue was used to stain mucus secretion by Calu-3. At days 3, 7, 14, 18, and 23, Calu-3 cells were washed with PBS, fixed with 4% paraformaldehyde and stained with alcian blue (1% *w*/*v* alcian blue in 3% *v*/*v* acetic acid/water at pH 2.5). Subsequently, the filter membranes were cut and mounted on glass slides using glycerol and sealed. The slides were imaged using an Olympus BX41 microscope (Olympus, Tokyo, Japan).

### 4.6. Confocal Microscopy

A549, Calu-3, 16-HBE and LFs were seeded on 35 mm glass bottom petri dish (Corning Costar, Lowell, MA, USA) at a density of 1.5 × 10^4^ cells. After 24 h, cells were incubated with LIP, LIP-HA4800 and LIP-HA14800 fluorescently labeled for 4 h at 37 °C. Next, cells were washed with PBS, fixed with 4% paraformaldehyde and DAPI solution was added to label the nuclei of cells.

To analyze the mucus penetration of different formulations of liposomes, we cultivated Calu-3 in ALI configuration to produce mucus. We then treated cells with LIP, LIP-HA4800, and LIP-HA14800 fluorescently labeled for 24 h at 37 °C. Cells were washed with PBS, fixed with 4% paraformaldehyde and DAPI solution was added to label the nuclei of cells.

Cells were observed by confocal laser microscopy Fluoview FV10i (Olympus, Tokyo, Japan). Regarding liposomes mucus penetration, cells were analyzed with a z-stack of 5 μm/slice.

### 4.7. Flow Cytometry for Liposomes Utake

A549, BOS-, and CTD-ILD-LFs were seeded on 12-well plates at a density of 2.5 × 10^4^ cells. After 24 h, cells were incubated with LIP, LIP-HA4800, and LIP-HA14800 fluorescently labeled for 4 h at 37 °C. Cells were then washed with PBS, harvested in cytometer tubes and analyzed by flow cytometer (Navios, Beckman Coulter, Brea, CA, USA) to quantify fluorescent signal.

Differentiated THP-1 cells seeded in 6-well culture plates (7 × 10^5^ cells), were exposed for 1 h to fluorescently labeled LIP, LIP-HA4800 and LIP-HA14800. Cells were also pre-incubated with 100× molar excess of free high MW HA (51,000 Da) for 1 h. Following medium removal, cells were washed twice with PBS, collected by using a cell scraper and finally re-suspended in 1 mL of PBS. Next, the intracellular uptake of liposomes was analyzed using a BD FACSCanto™ system (Becton Dickinson, Franklin Lakes, NJ, USA). Detection of fluorescent liposomes was carried out using FL-1 channel acquiring 10,000 events per samples, using Kaluza software and CellQuest software for Navios and Becton-Dickinson flow cytometers, respectively. Cells incubated in the absence of liposomes were used as control.

### 4.8. qRT-PCR

RNA extraction, complementary DNA synthesis, and qRT-PCR reactions were performed as previously described (Cannito et al. 2015). Human IL-1β, IL-12, TGF-β1, and VEGF-A mRNA levels were measured using the SYBR^®^ green method as described [38]. The amplification mix was prepared using Roche LightCycler FastStart DNA MasterPLUS SYBR Green I kit following manufacturer’s instructions and real-time PCR was performed using LightCycler instrument (Bio-Rad CFX Connect Thermocycler, Hercules, CA, USA). Oligonucleotide sequence of primers used for qRT-PCR were: sense, 5′-TGAAAGCTCTCCACCTCCAG-3′, ′reverse 5′-CACGCAGGACAGGTACAGAT-3′ (for human IL-1β); sense, 5′-AAGGAGGCGAGGTTCTAAGC-3′, reverse, 5′-AAGAGCCTCTGCTGCTTTTG-3′ (for human IL-12); sense, 5′-GGGACTATCCACCTGCAAGA-3′, reverse, 5′-CCTCCTTGGCGTAGTAGTCG-3′ (for human TGF-β1); sense, 5′-CCCACTGAGGAGTCCAACAT-3′, reverse, 5′-TTTCTTGCGCTTTCGTTTTT-3′ (for human VEGF-A). Gliceraldehyde-3-phosphate dehydrogenase (GAPDH, sense: 5′-TGGTATTCGGGAAGGACTCATGAC-3′, reverse: 5′-ATGCCAGTGAGCTTCCCGTTCAGC-3′) was used as internal reference and co-amplified with target samples using identical qRT-PCR conditions. Samples were run in triplicate and mRNA expression was generated for each sample. Specificity of the amplified PCR products was determined by melting curve analysis and confirmed by agarose gel electrophoresis.

### 4.9. Neutrophils and Monocytes Blood Isolation

Leucocytes were obtained from healthy blood donors collected at the Transfusion Center of San Matteo Hospital and monocytes were isolated by gradient centrifugation with Lympholyte^®^ (Cedarlane, Ontario, ON, Canada) in 50 mL conical tube and centrifuged for 30 min at 500 rcf without brake. Regarding monocytes, the peripheral blood mononuclear cell (PBMC) layer was carefully transferred into a new 50 mL conical tube and diluted with physiologic solution, centrifuged for 10 min decreasing the rcf of centrifugation to 200 rcf. Concerning neutrophils isolation, after cells blood separation with Lympholyte^®^, only the neutrophils layer was collected and washed with a physiologic solution and centrifuged at 400 rcf for 10 min. Cell pellet was lysed with VersaLyse^™^ (Beckman Coulter, Brea Beckman Coulte, Brea, CA, USA) for 20 min at room temperature to eliminate erythrocytes. Physiologic solution was added, and cells were centrifuged at 400 rcf for 10 min. This step was repeated until the erythrocytes were completely abolished.

### 4.10. Neutrophils Activation

1 × 10^6^ neutrophils derived from healthy donor blood were treated with LIP, LIP-HA4800, and LIP-HA14800 for 1 h at 37 °C. 2 μg mL^−1^ LPS was used as positive control. Afterwards, cells were labeled with antibodies against CD45-APC and CD11b-PE (Beckman Coulter, Brea, CA, USA) and analyzed with flow cytometry acquiring 10,000 events per sample, using Kaluza software (version 1.0, Beckman Coulter, Brea, CA, USA).

### 4.11. IL-8 and TGF-β Release From Monocytes

IL-8 and TGF-β production by monocytes was evaluated by ELISA technique. 2.5 × 10^5^ monocytes isolated from blood of healthy donors were incubated for 24 h with LIP, LIP-HA4800 and LIP-HA14800. Positive control cells were incubated with 1 μg mL^−1^ LPS. After incubations, surnatants were collected and centrifuged at 300 rcf to eliminate cells and debris. Next, surnatants were treated following the instructions of the manufacturers of Quantikine ELISA Human IL-8/CXCL8 Immunoassay kit (R&D system, Minneapolis, MN, USA) and Human TGF-β ELISA kit (Sigma-Aldrich, Milan, Italy). Obtained absorbance values were interpolated with the standard curve and expressed as mean ± standard deviation.

### 4.12. Statistical Analyses

Statistical differences between untreated cells and cells treated with liposomes were evaluated using one-way ANOVA analysis followed by Dunnett post hoc test for multiple comparison. All analyses were carried out with a GraphPad Prism 5.0 statistical program (GraphPad software, San Diego, CA, USA). A value *p* < 0.05 was considered statistically significant.

## Supplementary Materials

The following are available online at https://www.mdpi.com/1420-3049/24/18/3291/s1, Table S1: Characteristics of liposomal formulations, Table S2: Mean Fluorescence Intensity (MFI) of CD44 expression analyzed by flow cytometry, Figure S1: Confocal microscopy of 16-HBE and Calu-3, Figure S2: Pericellular coating and HA-fluorescent deposition, Figure S3: Alcian blue staining.
