# Supplementary material for: Hyaluronic Acid–Decorated Liposomes as Innovative Targeted Delivery System for Lung Fibrotic Cells
Source: Molecules. 2019 Sep 10;24(18):3291. doi: 10.3390/molecules24183291 (PMC6766933; doi:10.3390/molecules24183291)
Supplement: Supplementary file 1 [file molecules-24-03291-s001.pdf]

molecules

Supporting information

Setting the Basis to Use Hyaluronic Acid-Decorated Liposomes for Lung Fibrotic Disorders

Laura Pandolfi[1§], Vanessa Frangipane[1§], Claudia Bocca[2], Alessandro Marengo[3], Erika Tarro Genta[3], Sara Bozzini[1], Monica Morosini[1], Maura D'Amato[1], Simone Vitulo[1], Manuela Monti[4], Giuditta Comolli[5], Maria Teresa Scupoli[6,7], Elias Fattal[9], Silvia Arpicco[3]* and Federica Meloni[1,8]*

[1] Research Laboratory of lung diseases, section of cell biology, IRCCS Policlinico San Matteo Foundation, Pavia (Italy);
[2] Department of Clinical and Biological Sciences, University of Turin, Turin (Italy);
[3] Department of Drug Science and Technology, University of Turin (Italy);
[4] Laboratory of Biotechnology, Center of Regenerative medicine research, IRCCS San Matteo Foundation, Pavia (Italy);
[5] Experimental Research Laboratories, Biotechnology Area, IRCCS San Matteo Foundation, Pavia (Italy);
[6] Research Center LURM, Interdepartmental Laboratory of Medical Research, University of Verona, Verona (Italy)
[7] Department of Neurosciences, Biomedicine and Movement Sciences, University of Verona, Verona (Italy)
[8] Department of Internal Medicine, University of Pavia, Pavia (Italy)
[9] Institut Galien Paris-Sud, CNRS, Université Paris-Sud, Université Paris-Saclay, Châtenay-Malabry (France)

§ These authors contributed equally
*Correspondence: silvia.arpicco@unito.it Tel: +39 0116706668 (S.A.); f.meloni@smatteo.pv.it Tel.: +39 0382501017 (F.M.)

Table S1. Characteristics of Liposomal Formulations (means ± SD; n=3).

Formulation	Mean particle size (nm)	Polydispersity index	Zeta potential (mV)
LIP	207 ± 2.3	0.112	-27.2 ± 2.9
LIP-HA4800	221 ± 1.4	0.172	-10.1 ± 1.3
LIP-HA14800	245 ± 1.9	0.175	-24.6 ± 3.9

Table S2. Mean Fluorescence Intensity (MFI) of CD44 Expression Analyzed by Flow Cytometry.

Cell line	MFI
A549	55.35
Calu-3	14.53
16-HBE	2.55
BOS-LFs	56.72
CTD-ILD-LFs	77.71
THP-1	61.11
THP-1-PMA	74.46

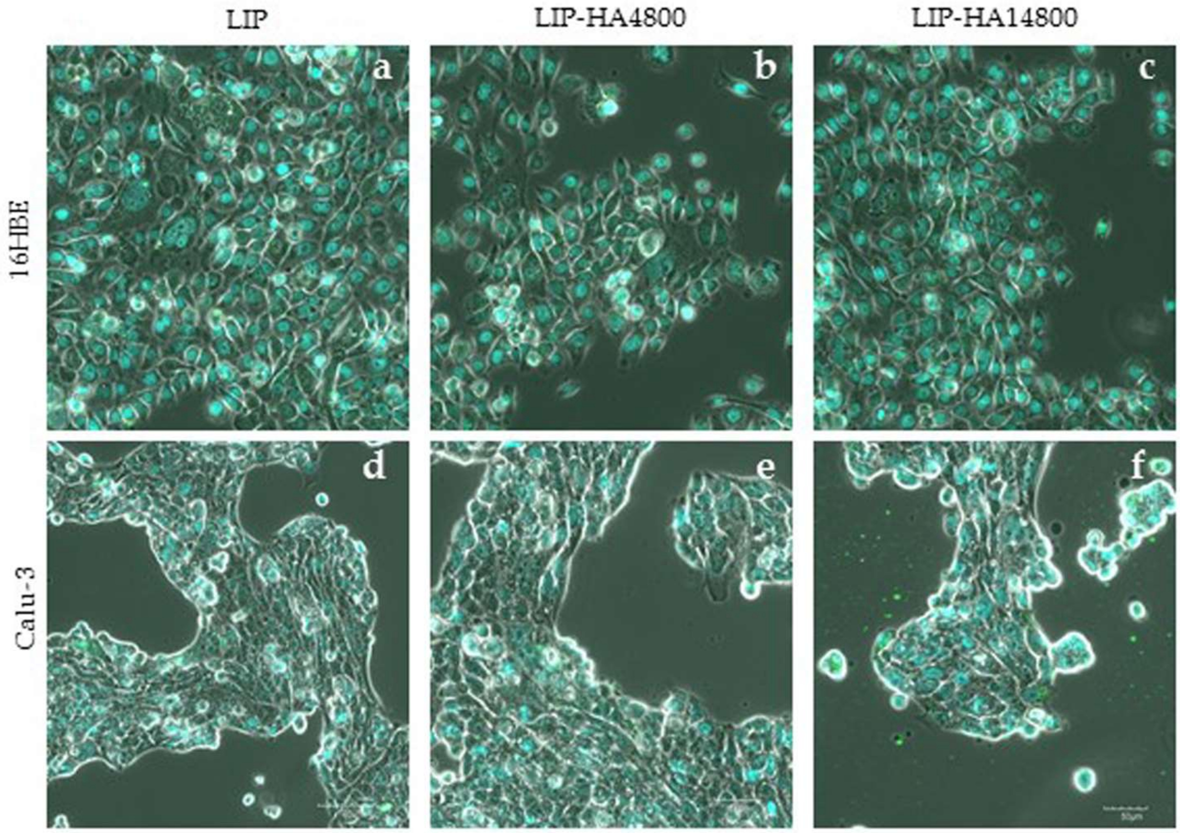

Figure S1. Liposomes internalization analyses by confocal microscopy on 16HBE (a-c) and Calu-3 (d-f) after 4 h of incubation. Scale bar = 50 µm.

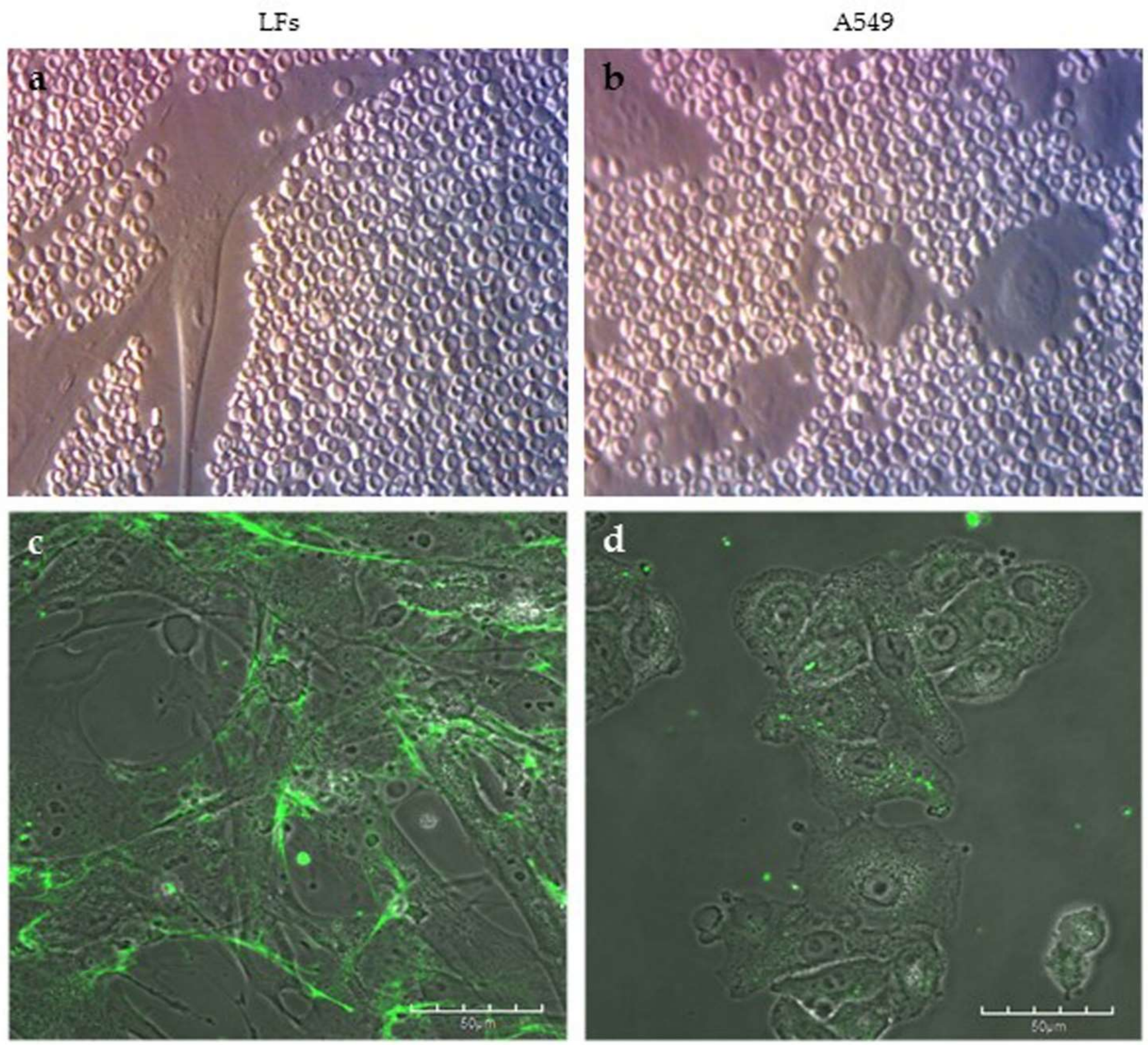

Figure S2. Pericellular coating (a,b) and fluorescent HA deposition (green signal, c,d) on LFs (a,c) and A549 (b,d). Scale bar = 50 µm.

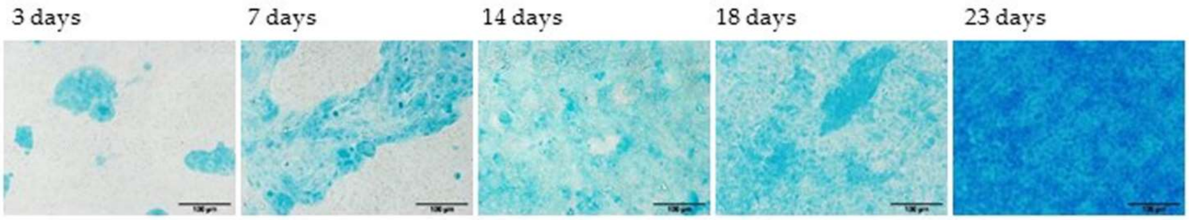

Figure S3. Alcian blue staining of mucus layer produced by Calu-3 cultivated in air-liquid interface configuration at different time points. Scale bar = 100 µm.
